# ChIP-Seq and RNA-Seq Reveal an AmrZ-Mediated Mechanism for Cyclic di-GMP Synthesis and Biofilm Development by *Pseudomonas aeruginosa*

**DOI:** 10.1371/journal.ppat.1003984

**Published:** 2014-03-06

**Authors:** Christopher J. Jones, David Newsom, Benjamin Kelly, Yasuhiko Irie, Laura K. Jennings, Binjie Xu, Dominique H. Limoli, Joe J. Harrison, Matthew R. Parsek, Peter White, Daniel J. Wozniak

**Affiliations:** 1 Department of Microbiology and Environmental Toxicology, University of California Santa Cruz, Santa Cruz, California, United States of America; 2 Department of Infection and Immunity and Center for Microbial Interface Biology, Ohio State University, Columbus, Ohio, United States of America; 3 Center for Microbial Pathogenesis, The Research Institute at Nationwide Children's Hospital, Columbus, Ohio, United States of America; 4 Department of Biology & Biochemistry, University of Bath, Claverton Down, Bath, United Kingdom; 5 Department of Microbiology, University of Washington, Seattle, Washington, United States of America; 6 Department of Microbiology, Ohio State University, Columbus, Ohio, United States of America; 7 Department of Biological Sciences, University of Calgary, Calgary, Alberta, Canada; University of Basel, Switzerland

## Abstract

The transcription factor AmrZ regulates genes important for *P. aeruginosa* virulence, including type IV pili, extracellular polysaccharides, and the flagellum; however, the global effect of AmrZ on gene expression remains unknown, and therefore, AmrZ may directly regulate many additional genes that are crucial for infection. Compared to the wild type strain, a *ΔamrZ* mutant exhibits a rugose colony phenotype, which is commonly observed in variants that accumulate the intracellular second messenger cyclic diguanylate (c-di-GMP). Cyclic di-GMP is produced by diguanylate cyclases (DGC) and degraded by phosphodiesterases (PDE). We hypothesized that AmrZ limits the intracellular accumulation of c-di-GMP through transcriptional repression of gene(s) encoding a DGC. In support of this, we observed elevated c-di-GMP in the Δ*amrZ* mutant compared to the wild type strain. Consistent with other strains that accumulate c-di-GMP, when grown as a biofilm, the Δ*amrZ* mutant formed larger microcolonies than the wild-type strain. This enhanced biofilm formation was abrogated by expression of a PDE. To identify potential target DGCs, a ChIP-Seq was performed and identified regions of the genome that are bound by AmrZ. RNA-Seq experiments revealed the entire AmrZ regulon, and characterized AmrZ as an activator or repressor at each binding site. We identified an AmrZ-repressed DGC-encoding gene (*PA4843*) from this cohort, which we named AmrZ dependent cyclase A (*adcA*). PAO1 overexpressing *adcA* accumulates 29-fold more c-di-GMP than the wild type strain, confirming the cyclase activity of AdcA. In biofilm reactors, a Δ*amrZ* Δ*adcA* double mutant formed smaller microcolonies than the single Δ*amrZ* mutant, indicating *adcA* is responsible for the hyper biofilm phenotype of the Δ*amrZ* mutant. This study combined the techniques of ChIP-Seq and RNA-Seq to define the comprehensive regulon of a bifunctional transcriptional regulator. Moreover, we identified a c-di-GMP mediated mechanism for AmrZ regulation of biofilm formation and chronicity.

## Author Summary

Pathogenic bacteria such as *Pseudomonas aeruginosa* utilize a wide variety of systems to sense and respond to the changing conditions during an infection. When a stress is sensed, signals are transmitted to impact expression of many genes that allow the bacterium to adapt to the changing conditions. AmrZ is a protein that regulates production of several virulence-associated gene products, though we predicted that its role in virulence was more expansive than previously described. Transcription factors such as AmrZ often affect the expression of a gene by binding and promoting or inhibiting expression of the target gene. Two global techniques were utilized to determine where AmrZ binds in the genome, and what effect AmrZ has once bound. This approach revealed that AmrZ represses the production of a signaling molecule called cyclic diguanylate, which is known to induce the formation of difficult to treat communities of bacteria called biofilms. This study also identified many novel targets of AmrZ to promote future studies of this regulator. Collectively, these data can be utilized to develop treatments to inhibit biofilm formation during devastating chronic infections.

## Introduction

*Pseudomonas aeruginosa* is a Gram-negative opportunistic pathogen that is a major burden on the health care industry. Up to 10% of all nosocomial infections are attributed to *P. aeruginosa*, with mortality rates approaching 40% in patients with bacteremia [1], [2]. This bacterium is often a causative agent of sepsis, as well as acute and chronic infections of the airway, burn wounds, skin, and medical devices such as catheters [1], [3].

Additionally, *P. aeruginosa* forms biofilms that contribute significantly to disease [4]. The formation of a biofilm by *P. aeruginosa* confers resistance to antibiotic treatment and immune cells [5]–[7]. The classical definition of a biofilm involves a community of bacteria adhered to a surface encased in a self-produced matrix [3], [8]–[11]. *P. aeruginosa* forms these biofilms in the environment, on implanted devices such as catheters, and in wound infections [12]. In addition, *P. aeruginosa* forms biofilms suspended in the dehydrated pulmonary mucus plugs of cystic fibrosis patients [13], [14]. Biofilms are often recalcitrant to antibiotics, have anti-phagocytic properties, and are difficult to treat, commonly accounting for the persistence of chronic infections [7], [15]–[17].

Our laboratory has identified the ribbon-helix-helix transcription factor AmrZ (alginate and motility regulator Z) as a modulator of *P. aeruginosa* biofilm development and virulence [18], [19]. Five AmrZ-regulated virulence factors have been identified through targeted molecular approaches; however, the global effect of AmrZ on expression of *P. aeruginosa* genes is unknown. AmrZ directly represses transcription of *fleQ* and thus motility [20], [21], and its own transcription in a feedback loop [22], [23]. Additionally, AmrZ inhibits production of the extracellular polysaccharide Psl by repressing transcription of the *psl* operon [19]. In contrast, AmrZ activates alginate production by binding the *algD* promoter [24], [25] and is essential for twitching motility and formation of a type IV pilus [26]. Each of these AmrZ-regulated genes have been linked to biofilms and *P. aeruginosa* pathogenicity. The major limitation of the previous approaches is that they are biased towards genes that produce an easily observed phenotype, potentially overlooking many AmrZ-regulated genes that are important in infection. Here, we present a systems-level analysis of the AmrZ regulon utilizing ChIP-Seq and RNA-Seq [27], [28]. By combining these two high-throughput techniques, the genome can be scanned for functional AmrZ binding sites. Additionally, these data allow classification of members of the AmrZ regulon into activated or repressed promoters, as well as direct vs. indirect regulation. Herein, we identified 398 regions of the genome bound by AmrZ (≥3-fold enrichment). The RNA-Seq identified 333 genes that were differentially expressed when comparing a Δ*amrZ* mutant to a complemented strain (≥2-fold difference). Comparison of AmrZ-bound and AmrZ-regulated genes identified 9 genes directly activated by AmrZ and 49 genes that were directly repressed. Many of these genes have been implicated in pathogenesis, highlighting the importance of AmrZ in *P. aeruginosa* virulence. Finally, these data allow comparisons of the sequence specificity of AmrZ bound promoters, further defining the consensus AmrZ binding site and lending insight into the mechanism of regulation by AmrZ.

One AmrZ-dependent pathway was investigated in detail since it provided important insights into earlier findings that Δ*amrZ* mutants form hyper biofilms compared with the parental strain, PAO1 [19]. The present study provides a molecular basis for this finding since we discovered that AmrZ directly represses a predicted diguanylate cyclase-encoding gene (*PA4843*), which we named *adcA* (AmrZ dependent cyclase A,). Repression of *adcA* led to reduced amounts of the second messenger c-di-GMP. This regulation event explains the hyper- aggregative and -biofilm phenotype of a Δ*amrZ* strain, as elevated c-di-GMP is often associated with the rugose small colony variant phenotype that shares these characteristics [29]–[31]. Recent reports indicate that reducing c-di-GMP in *P. aeruginosa* biofilm infections leads to biofilm dissolution [32], [33]. Regulation of c-di-GMP by AmrZ could lend insights into the establishment and persistence of chronic *P. aeruginosa* infections and open novel avenues of treatment.

## Results

### *amrZ* mutants have an RSCV phenotype

**Figure 1:**
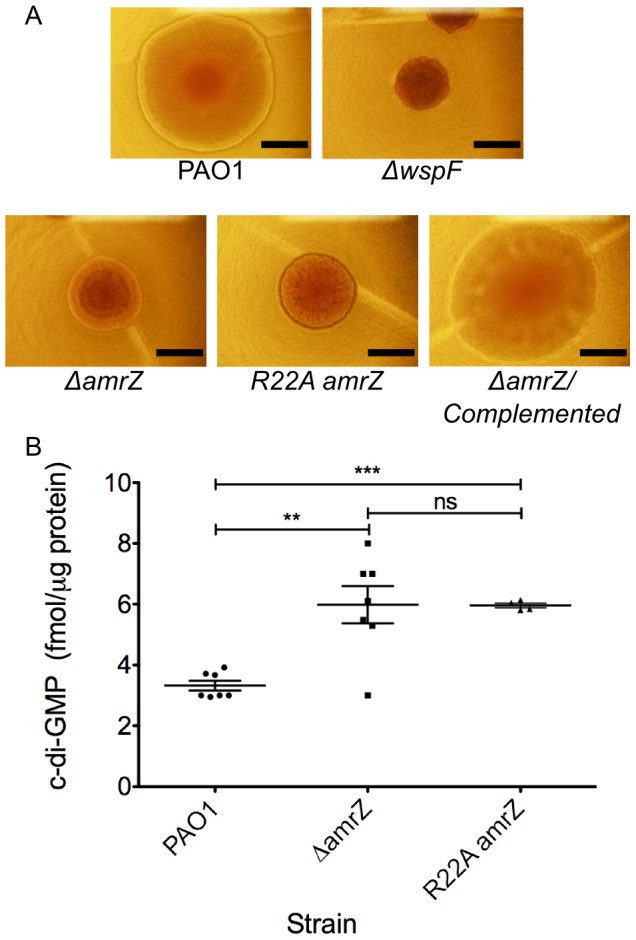
*amrZ* mutants show characteristics of Rugose Small Colony Variant Strains. **A Δ*amrZ* mutants have RSCV phenotype.** Images represent colony morphology after overnight growth on VBMM agar. RSCV phenotype was observed in Δ*amrZ* and R22A DNA-binding deficient *amrZ* mutants but not in the parental PAO1. Chromosomal complementation of *amrZ* reverts the colony morphology to that of the smooth parental PAO1. Δ*wspF* is included as a known RSCV strain. Scale bars indicate 1 mm. **B Δ** ***amrZ*** **mutants exhibit increased intracellular c-di-GMP.** Direct LC-MS/MS measurement of c-di-GMP in plate-grown bacteria indicates that a Δ*amrZ* mutant accumulates significantly more intracellular c-di-GMP than the wild-type PAO1. The DNA binding deficient R22A AmrZ mutant has elevated c-di-GMP. Individual measurements are displayed, along with mean and standard error of the mean. Statistical significance was determined using Student's t-test. (** p≤0.01, *** p≤0.001).

Upon observation of overnight growth on VBMM, the wild-type strain PAO1 formed a smooth colony, while the Δ*amrZ* mutant formed an aggregated rugose small colony variant (RSCV) morphology. (Figure 1A). Prevention of rugosity was dependent on AmrZ binding DNA, as the DNA binding deficient R22A AmrZ mutant is also an RSCV (Figure 1A). Chromosomal complementation of the Δ*amrZ* mutant relieves the rugose phenotype and returns the colony morphology to that of the smooth parental strain (Figure 1A). We have included the well-defined RSCV Δ*wspF* for comparison [29], [34], [35]. The RSCV phenotype of the Δ*wspF* mutant has been attributed to the loss of repression of the diguanylate cyclase WspR, leading to elevated intracellular c-di-GMP [32], [36]. Cyclic di-GMP modulates the activity of the transcriptional regulator FleQ at the *pel* locus, switching FleQ from a repressor to an activator [37], [38]. Psl and Pel polysaccharide overproduction in these strains is responsible for the hyper aggregative phenotype and rugose colony morphology observed [35]. We therefore hypothesized that the Δ*amrZ* mutant displayed a RSCV phenotype due to elevated intracellular c-di-GMP. To test this, we purified nucleotide pools from plate-grown cells and measured the c-di-GMP via LC-MS/MS (Figure 1B) [39]. We observed that the Δ*amrZ* mutant accumulated nearly double the intracellular c-di-GMP compared to parental wild type PAO1 (p≤0.01). A two-fold change in c-di-GMP levels can have drastic effects on cell physiology and biofilm formation [39]–[42]. These data are consistent with our classification of Δ*amrZ* mutants as RSCV. Additionally, we observed that the DNA binding deficient R22A *amrZ* mutant had similar intracellular levels of c-di-GMP as the Δ*amrZ* strain (data not shown), indicating that the AmrZ contribution to low c-di-GMP is DNA binding dependent. This observation, in combination with elevated c-di-GMP in the *amrZ* mutants suggests that AmrZ-mediated modulation of c-di-GMP is either through transcriptional repression of a diguanylate cyclase or activation of a phosphodiesterase. Since AmrZ is a bifunctional transcriptional regulator [22], [24], [25], either of these mechanisms is possible. Therefore, to provide a comprehensive analysis of the AmrZ regulon and to define the mechanistic basis for c-di-GMP accumulation in the Δ*amrZ* mutant, RNA-Seq and ChIP-Seq strategies were undertaken.

### ChIP-Seq provides an unbiased analysis of AmrZ binding to genomic DNA

Previous studies identified four AmrZ-bound promoters utilizing standard molecular methods such as DNA footprinting and Electrophoretic Mobility Shift Assays (EMSA) [18], [19], [22]–[26], [43]. Though these methods are recognized as the standard for DNA binding analysis, we wished to perform a genome-wide screen for AmrZ binding sites. Chromatin immunoprecipitation (ChIP) allows us to purify DNA bound AmrZ directly from cells [27], [28], [44], [45]. In this assay, chromatin bound AmrZ was cross-linked, the DNA sheared, nonspecific proteins and nucleic acids removed, and the DNA was purified and quantified using high-throughput parallel DNA sequencing. The resulting ChIP-Seq tags were analyzed using HOMER (Hypergeometric Optimization of Motif EnRichment) a suite of tools for ChIP-Seq analysis and motif discovery [46]. This generated a complete map of genomic areas to which AmrZ binds (Table S1). Conditions were optimized by using previously studied positive control DNA (*algD*, *amrZ*) and a negative control region (*algB*) [22], [24], [25]. Consistent with the literature, *algD* and *amrZ* promoters were significantly enriched over input DNA (6.68 and 4.80 fold, respectively), while the *algB* promoter demonstrated no significant enrichment compared to input DNA. The previously published AmrZ interaction at the *fleQ* and *pslA* promoters was also confirmed with this data set (Table 1), indicating the stringency of the analysis. The relatively low enrichment of these two previously described promoters by AmrZ provides a reference to which other interactions can be compared, suggesting that the interactions described here (≥3-fold enrichment) are biologically significant in the cell. In total, we identified 398 regions of the genome that were bound by AmrZ (≥3-fold enrichment over input) (Table S1).

**Table 1:** RNA-Seq indicates AmrZ represses pyochelin and pyoverdine siderophore systems.

Gene Name	Gene ID	Gene Product	RNA-Seq fold Change	p-value
**Pyochelin synthesis genes**				
*pchC*	PA4229	pyochelin biosynthetic protein PchC	−8.91	0.00000
*pchG*	PA4224	pyochelin biosynthetic protein PchG	−7.13	0.00000
*pchB*	PA4230	isochorismate-pyruvate lyase	−5.90	0.00000
*pchF*	PA4225	pyochelin synthetase	−5.09	0.00000
*pchD*	PA4228	pyochelin biosynthesis protein PchD	−4.25	0.00000
*pchA*	PA4231	salicylate biosynthesis isochorismate synthase	−4.17	0.00000
*pchE*	PA4226	dihydroaeruginoic acid synthetase	−4.02	0.00000
*pchR*	PA4227	transcriptional regulator PchR	−2.54	0.00000
**Pyochelin receptor gene**				
*fptA*	PA4221	Fe(III)-pyochelin outer membrane receptor	−5.44	0.00000
**Pyoverdine synthesis genes**				
*pvdA*	PA2386	L-ornithine N5-oxygenase	−2.94	0.00000
*pvdQ*	PA2385	3-oxo-C12-homoserine lactone acylase PvdQ	−1.99	0.00000
*pvdP*	PA2392	protein PvdP	−1.91	0.00000
*pvdH*	PA2413	diaminobutyrate–2-oxoglutarate aminotransferase	−1.76	0.00038
*pvdS*	PA2426	extracytoplasmic-function sigma-70 factor	−1.65	0.00116
*pvdL*	PA2424	peptide synthase	−1.57	0.00000
*pvdR*	PA2389	protein PvdR	−1.53	0.00095
*pvdN*	PA2394	protein PvdN	−1.50	0.00244
*pvdJ*	PA2400	protein PvdJ	−1.40	0.00014
*pvdD*	PA2399	pyoverdine synthetase D	−1.39	0.00014
*pvdF*	PA2396	pyoverdine synthetase F	−1.37	0.00347
*pvdT*	PA2390	protein PvdT	−1.35	0.01248
*pvdE*	PA2397	pyoverdine biosynthesis protein PvdE	−1.33	0.00364
**Pyoverdine receptor genes**				
*fpvA*	PA2398	ferripyoverdine receptor	−2.01	0.00000
*fpvB*	PA4168	second ferric pyoverdine receptor FpvB	−1.45	0.00038
**Pyoverdine regulator gene**				
*ppyR*	PA2663	psl and pyoverdine operon regulator, PpyR	−1.97	0.00000

**Figure 2:**
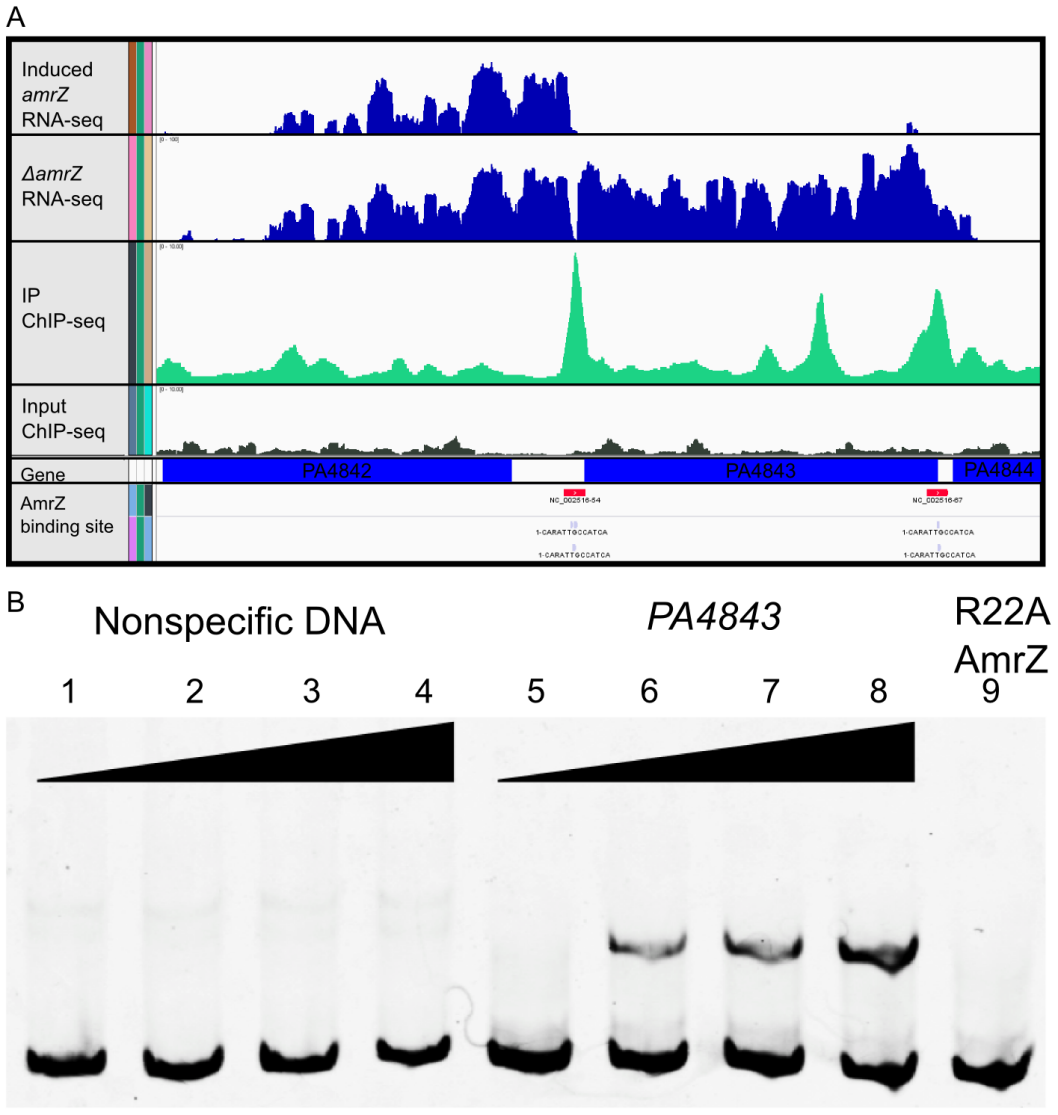
Analysis of ChIP-Seq and RNA-Seq data identifies AmrZ regulon. A Representative images of the Integrated Genome Viewer software utilized to analyze sequencing data. Histograms comparing RNA sequence reads of arabinose-induced *amrZ* to Δ*amrZ* mutant strains are colored blue. Histograms depict ChIP-Sequence reads for arabinose induced *amrZ* (green) and Δ*amrZ* mutant strains (grey). AmrZ binding (red) is indicated in the promoter of *PA4843* and *PA4844* by observing ≥3 fold more sequence reads in the induced AmrZ ChIP sample compared to the Δ*amrZ* mutant. B AmrZ specifically binds to the *PA4843* promoter. A 168 bp DNA fragment was amplified from the *PA4843* promoter containing the AmrZ binding site discovered by ChIP-seq and labeled with 6FAM at the 5′ end. Interaction between this fragment and purified AmrZ was measured by EMSA (Electrophoretic Mobility Shift Assay; lanes 5–9). Each reaction contains 5 nM of FAM-labeled DNA, and increasing concentrations of AmrZ. A 174 bp DNA sequence within the *algD* coding sequence but lacking an AmrZ binding site was selected as the specificity control (lanes 1–4). The AmrZ concentration for each lane is as below. Lanes 1–8 contain wild type AmrZ. Lane 1 and 5, 0 nM; lanes 2 and 6, 100 nM; lanes 3 and 7, 200 nM; lanes 4 and 8, 400 nM. Lane 9 contains 400 nM of purified AmrZR22A (DNA binding deficient AmrZ).

Output for one significantly enriched region is included for reference (Figure 2A). In this example, AmrZ binds upstream of the *PA4843* gene in the immunoprecipitated sample (green histogram), however, this enrichment is not present in the input sample (grey histogram). Other regions of the genome that were bound by AmrZ appeared similar. Specific AmrZ binding to the *PA4843* promoter region was confirmed using an Electrophoretic Mobility Shift Assay (Figure 2B).

### AmrZ consensus binding site is defined

**Figure 3:**
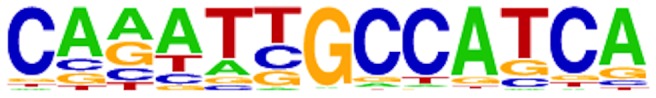
ChIP-Seq reveals the AmrZ consensus-binding site. Depiction of the consensus AmrZ binding site obtained by analyzing all AmrZ-enriched sequences. Consensus sequence contains an A/T rich region followed by GCC, followed by an A/T rich region. This sequence was observed in 54.7% of AmrZ-bound DNA, with a p-value of 1e-120. Motif analysis was performed utilizing tools within the HOMER analysis package.

The ChIP-Seq analysis allows one to predict consensus-binding sites based on identification of common sequences within enriched DNA. Based on these analyses we defined a consensus AmrZ binding site (Figure 3). The 13 nt motif was present in 54.7% of all enriched DNA fragments, but in only 10.93% of background reads, producing a significant enrichment of this sequence (p = 1e–120). This motif resembles that reported elsewhere for AmrZ binding using DNA binding and mutagenesis studies [18], [23]–[26], [43]. This motif is also contained in the crystal structure of AmrZ bound to the *amrZ1* binding site identified by Pryor *et al.* [23]. Putative AmrZ binding sites were assigned to a selection of the AmrZ-enriched DNA fragments based on these consensus sequences and analysis of ChIP-Seq reads.

### Transcriptional profiling via RNA-Seq defines the AmrZ regulon

Previous work demonstrated that AmrZ regulates genes in a variety of pathways, many of which are implicated in virulence [19], [21], [25], [26]. However, the extent of the AmrZ regulon is unknown. RNA-Seq allows comparison of sequences of the total mRNA from a Δ*amrZ* mutant to a complemented strain, elucidating the effect of AmrZ on all genes in the cell, both positive and negative. Total RNA was isolated from a mid-exponential culture (OD_600_ 0.5±0.1) of a Δ*amrZ* mutant containing the empty pHERD20T vector and a complemented strain containing the arabinose inducible AmrZ expression vector pCJ3. These growth conditions were chosen to match those utilized in the ChIP-Seq experiment. cDNA was synthesized and the resulting product was tagged and quantified using high-throughput parallel DNA sequencing. mRNA expression levels and differential expression analysis was performed using the Bioconductor package *DEseq* [47]. Three hundred and thirty eight genes were significantly regulated at least 2-fold (Benjamini-Hochberg adjusted p value <0.05), with 89 genes activated- and 249 genes repressed- by AmrZ (Table S2). Several of the AmrZ-regulated genes described in the literature were identified in this analysis, including *algD* (activated by AmrZ 19.74 fold) and *fleQ* (repressed by AmrZ 8.05 fold).

The RNA-Seq data indicate that AmrZ strongly, though indirectly, represses many genes involved in iron acquisition, suggesting a novel mechanism for AmrZ mediated control of virulence (Table 1). AmrZ significantly repressed many genes in the pyochelin and pyoverdine synthesis operons, including *ppyR*. In addition, the Fe(III)-pyochelin receptor *fptA* and ferripyoverdine receptors *fpvA* and *fpvB* were all significantly repressed by AmrZ, (5.44, 2.01, and 1.45 fold, respectively), suggesting that reliance on the iron acquisition systems is reduced in strains where AmrZ is highly expressed, such as in mucoid isolates from the CF lung. Previous reports indicate that the iron concentration in the CF sputum and lung is elevated [48]-[50], supporting the hypothesis that there is sufficient iron in the CF lung for bacterial growth with reduced dependence on the high-affinity iron acquisition systems. Many virulence factors are iron-regulated, so the impact of AmrZ-mediated siderophore repression may contribute significantly to the establishment of chronic infections [51], [52]. There was no alteration of the transcription of the iron-dependent master regulator Fur in the Δ*amrZ* mutant, implying that AmrZ regulates these iron acquisition genes independent of Fur, perhaps through small RNAs or downstream members of the Fur regulon that have yet to be identified. Future studies will explore the relationship of AmrZ and iron acquisition during infection.

### AmrZ directly regulates many genes associated with virulence

The results from the RNA-Seq and ChIP-Seq were further evaluated to determine genes potentially directly regulated by AmrZ. To accomplish this, the list of AmrZ-bound genomic regions (at least 3-fold enrichment) was filtered using the list of target genes regulated by AmrZ as determined by the RNA-Seq (at least 2-fold regulation). This approach allows classification of genes based on AmrZ binding status and AmrZ-mediated regulation. Interestingly, only 9 of the AmrZ-activated and 49 of the AmrZ-repressed genes were identified in the ChIP-Seq as also containing an AmrZ binding site within 500 base pairs of the start of the coding region of the gene (Table 2), suggesting that there are 80 activated and 200 repressed genes with promoters that were not directly bound by AmrZ, suggesting indirect regulation. One AmrZ directly activated gene is *algD*, a known AmrZ-dependent gene [24]. Other AmrZ-activated genes in Table 2A include a putative alginate lyase and members of the *pel* operon. These two genes, in combination with activation of the *algD* operon, suggest that when expressed, AmrZ affects the *P. aeruginosa* polysaccharide profile. Additionally, AmrZ directly activates the cyclic di-GMP response gene *cdrA*, which is correlated with polysaccharide overexpression [30]. Table 2B depicts genes directly repressed by AmrZ. In addition to the previously described *fleQ*, this list includes many genes that are known or predicted to be involved in virulence including: pyochelin synthesis (*pchG*), aggregation (*siaA*), flagellum synthesis (*fleQ, flgG, flgE, fliF*), alternative type IV pili production (*flp*), chemotaxis (*PA2867, pctC, PA4844*), multidrug transport (*PA3401*), and rhamnolipid production and quorum sensing (*rhlR*). Several of the directly AmrZ-repressed genes are predicted to be involved in Type VI secretion: *PA1657, PA1664, PA1668*. Type VI secretion is a recently-described system that is involved in *P. aeruginosa* pathogenesis and fratricide [53]–[56]. Specifically, the Type VI genes repressed by AmrZ belong to the HSI-II locus, which is involved in *P. aeruginosa* pathogenicity. HSI-II mutant strains exhibit a delay in mortality in both murine lung and burn wound infections [54]. This regulation may contribute to the role of AmrZ during infection.

**Table 2:** Systems-level analysis of the AmrZ regulon.

A			
Nearest Gene ID	RNA-Seq Fold Change	ChIP-Seq Fold Enrichment	Gene Product
*amrZ*	483.31	4.80	alginate and motility regulator Z
*PA1784*	19.74	5.59	hypothetical protein-(homologous to Klebsiella alginate lyase)
*pelB*	6.64	5.76	PelB
*PA0102*	4.57	3.05	carbonic anhydrase
*algD*	3.79	6.68	GDP-mannose 6-dehydrogenase AlgD
*PA1069*	3.22	3.58	hypothetical protein
*PA0082*	2.84	7.94	TssA1 Type VI secretion
*PA4625*	2.84	4.24	CdrA
*vreA*	2.67	6.39	VreA

B			
Nearest Gene ID	RNA-Seq Fold Change	ChIP-Seq Fold Enrichment	Gene Product
*PA4843*	−40.27	4.48	two-component response regulator
*PA2867*	−14.89	9.60	chemotaxis transducer
*PA1913*	−13.51	5.01	hypothetical protein (no prediction)
*PA3691*	−11.66	3.31	hypothetical protein (no prediction)
*PA5138*	−10.23	4.20	predicted ABC-type amino acid transport/signal transduction
*PA3235*	−9.18	6.49	predicted membrane protein
*PA4222*	−9.09	7.13	ABC transporter ATP-binding protein
*PA4677*	−8.96	5.99	predicted dipeptidase
*fleQ*	−8.05	3.00	transcriptional regulator FleQ
*PA3234*	−7.63	3.88	acetate permease
*pchG*	−7.13	13.60	pyochelin biosynthetic protein PchG
*PA0172*	−6.59	11.93	SiaA SDS induced aggregation
*PA5167*	−5.93	4.56	c4-dicarboxylate-binding protein
*PA1657*	−5.31	3.72	HsiB2 (predicted Type VI secretion)
*PA0038*	−5.12	7.13	hypothetical protein (no prediction)
*PA3722*	−5.11	3.30	hypothetical protein (no prediction)
*PA1941*	−4.87	3.28	hypothetical protein (no prediction)
*PA4324*	−4.75	4.77	predicted PilZ domain
*PA1680*	−4.56	6.48	predicted 3-oxoadipate enol-lactonase activity
*PA0938*	−4.43	3.53	hypothetical protein (predicted exopolysaccharide chain length determinant protein)
*flgG*	−4.25	3.47	flagellar basal body rod protein FlgG
*PA1199*	−4.04	4.33	lipoprotein
*PA2462*	−3.75	4.89	hypothetical protein (contains type 1 export signal)
*pctC*	−3.47	10.67	chemotactic transducer PctC
*PA4683*	−3.32	4.87	hypothetical protein (no prediction)
*flp*	−3.17	7.12	type IVb pilin, Flp
*flgE*	−3.12	4.89	flagellar hook protein FlgE
*PA4844*	−3.1	4.69	chemotaxis transducer
*PA3367*	−3.02	3.17	hypothetical protein (contains type 1 export signal)
*PA1340*	−3.01	5.87	amino acid ABC transporter membrane protein
*PA3951*	−2.91	3.72	hypothetical protein (no prediction)
*PA3413*	−2.90	3.30	predicted SOS response gene
*rhlR*	−2.88	5.57	transcriptional regulator RhlR
*PA1664*	−2.86	5.77	OrfX (secreted by Type VI)
*PA4033*	−2.75	5.32	MucE
*PA0494*	−2.73	8.98	acetyl-CoA carboxylase biotin carboxylase subunit
*PA3353*	−2.73	4.04	predicted c-di-GMP binding protein
*PA0472*	−2.61	3.51	RNA polymerase sigma factor FiuI, sigma 24 homolog
*PA1088*	−2.56	4.94	Predicted magnesium protoporphyrin IX methyltransferase activity
*PA2567*	−2.50	3.63	predicted phosphodiesterase
*PA3401*	−2.41	8.88	predicted ABC-type multidrug transport system, permease component
*fliF*	−2.38	5.49	flagellar MS-ring protein
*phzA1*	−2.29	5.24	phenazine biosynthesis protein
*ygbB*	−2.15	3.65	2-C-methyl-D-erythritol 2,4-cyclodiphosphate synthase
*PA3019*	−2.09	17.74	ABC transporter ATP-binding protein
*PA4980*	−2.09	7.55	enoyl-CoA hydratase
*PA1939*	−2.07	3.96	predicted OLD family endonuclease
*PA1668*	−2.04	6.17	DotU2 Type VI
*PA1738*	−2.00	3.56	transcriptional regulator

ChIP-Seq identifies regions of the genome bound by AmrZ (≥3-fold enrichment), while transcriptional profiling via RNA-Seq identifies differential regulation at these genes (≥2-fold regulation). **A** Table indicates genes directly activated by AmrZ. **B** Table indicates genes directly repressed by AmrZ.

Another group of AmrZ directly repressed genes are those predicted to be involved in cyclic diguanylate signaling. These include a predicted diguanylate cyclase (*PA4843*), a predicted phosphodiesterase (*PA2567*), and hypothetical proteins that are proposed c-di-GMP effector proteins containing PilZ domains (*PA4324, PA3353*). *PA4324* does not appear to be part of an operon, while *PA3353* is in the *flgM* operon and may have a function in flagella motility [57]. Dysregulation of c-di-GMP signaling could account for the hyper-aggregative phenotype of a Δ*amrZ* mutant. We explore this system further in this study.

### A common mechanism of AmrZ-mediated repression

**Figure 4:**
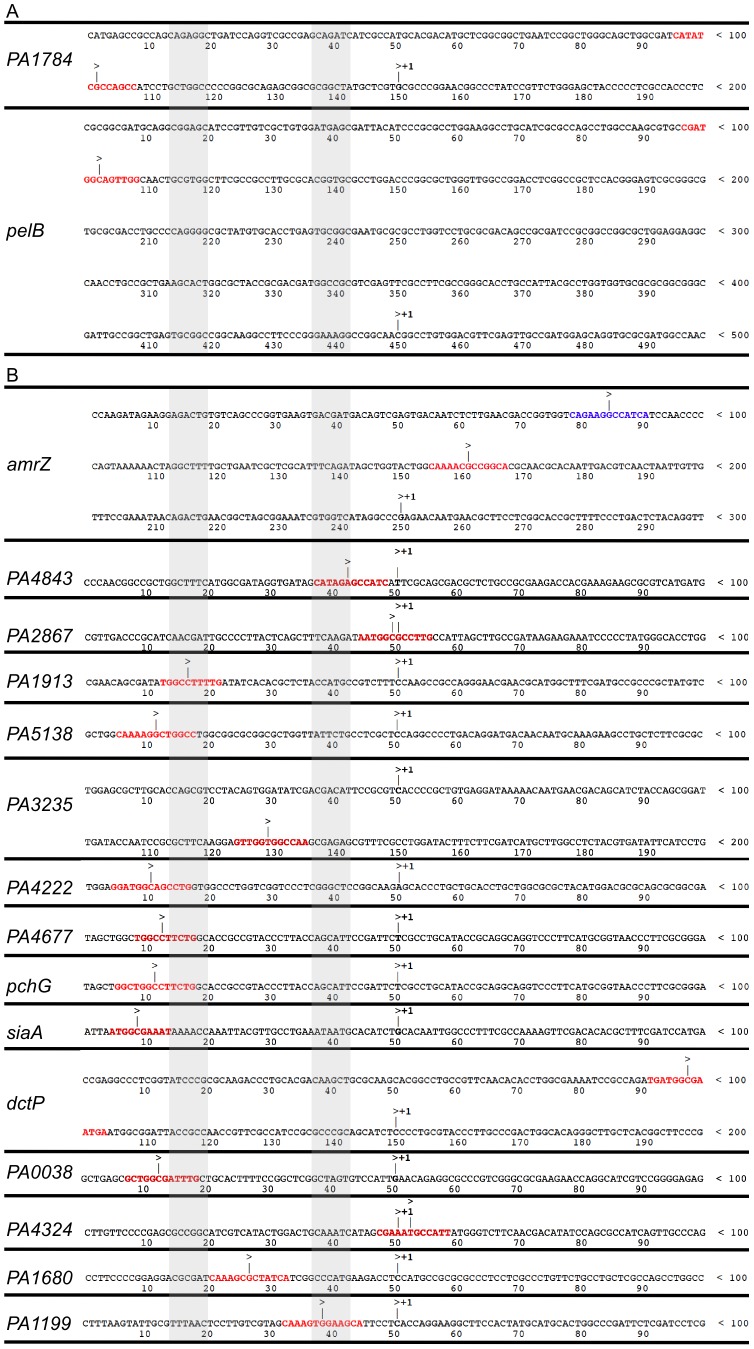
AmrZ binds near the start of transcription of repressed genes, but not activated genes. Promoter alignment of genes bound by AmrZ (≥4 fold enrichment) and **A** activated or **B** repressed (≥4 fold regulation). Alignment of repressed genes implies a common mechanism for AmrZ-mediated repression. Transcriptional start sites were called from RNA-Seq analysis. Sequences of AmrZ-repressed promoters are aligned at their transcriptional start site (+1). The putative AmrZ binding site is indicated by red text. Blue text in the *amrZ* promoter indicates a second AmrZ binding site, previously annotated AmrZ2. The promoter regions are indicated by grey shading.

Transcriptional start sites were obtained from RNA-Seq data by observing where the sequence reads begin upstream of a coding region [58]. By performing this analysis to a selection of directly AmrZ-regulated promoters, the proximity of the AmrZ binding site was observed relative to the transcription start site. Promoters with strong AmrZ binding (≥4-fold enrichment) and regulation (≥4-fold regulation) were chosen for an alignment of the AmrZ binding site to the start of transcription. The two strongly activated promoters did not suggest a common mechanism (Figure 4A). However, with the exception of *PA3235*, each of the directly AmrZ-repressed promoters observed contained an AmrZ binding site from −100 to +15 relative to the transcription start site (Figure 4B). This implies that during repression, AmrZ interferes with the binding of RNA polymerase to the promoter, a common mechanism of bacterial transcriptional repression.

Previous publications have identified two AmrZ binding sites in the *amrZ* promoter, *amrZ1* and *amrZ2* [22], [23]. The *amrZ1* binding site was identified by the ChIP-Seq (Figure 4B, red binding site). The previously identified *amrZ2*-binding site was not specifically identified by ChIP-Seq, however, this is likely due to the reduced AmrZ affinity for the *amrZ2* binding site [18], [22]. Analysis of the read alignment of the immunoprecipitated sample reveals a biphasic peak including both the *amrZ1* and *amrZ2* binding sites. One gene (*PA3235*) that was repressed by AmrZ lacked a binding site in the promoter. However, AmrZ did bind 70 bp downstream of the observed *PA3235* start of transcription. This may indicate a second mechanism of AmrZ repression, where bound AmrZ interferes with the elongation of the transcript.

Analysis of the proximal promoter regions of AmrZ-regulated genes indicates that AmrZ may affect RNA polymerase assembly directed by several sigma factors. For example, the −10 and −35 boxes of *siaA* appear to indicate that this promoter is RpoD-dependent (−35TTGaCc/−10TAtAAT), while the promoter of *PA4843* appears to match the consensus sequence for a σ^N^ dependent promoter (−24GG/−12GC) [59]. There was no discernable pattern in the relation of the AmrZ binding site to the start of transcription in the AmrZ-activated genes, indicating that there may be several mechanisms of AmrZ-mediated direct activation.

### *adcA (PA4843)* encodes a diguanylate cyclase

**Figure 5:**
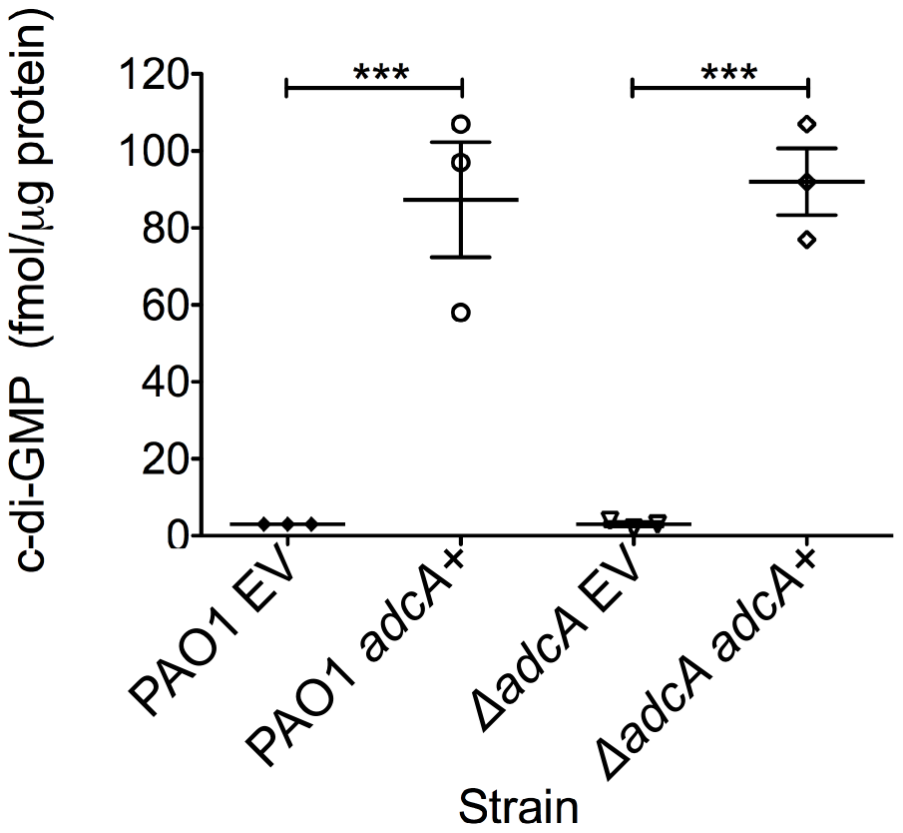
*PA4843* (*adcA*) encodes diguanylate cyclase. Expression of *adcA* increases intracellular c-di-GMP. *adcA* was cloned into an arabinose-inducible vector and strains were grown on LANS plates with 0.5% arabinose overnight. Direct LC-MS/MS measurement of c-di-GMP revealed that both PAO1 and Δ*adcA* containing the empty vector (EV) accumulated minimal amounts of c-di-GMP, while induction of *adcA* (*adcA+*) was correlated with high accumulation of intracellular c-di-GMP. Each graph point represents the average of three biological replicate performed in triplicates. Significance determined using Student's *t*-test (*** p≤0.001)

The gene most highly repressed by AmrZ was *PA4843* (40-fold) (Table 2B). Predictions based on the structure and function of PleD from *Caulobacter crescentus* indicates that *PA4843* contains two component receiver domains (Rec), an I-site, and a GGEEF cyclase domain (Figure S1). Previously, *PA4843* was described as a putative diguanylate cyclase [60] since it contains a conserved cyclase domain; however, no reports demonstrate functional cyclase activity for the *PA4843*-encoding gene. Additionally, deletion of this gene in strain PA14 did not impact attachment or host cell cytotoxicity [60]. Because *PA4843* was the most highly repressed AmrZ target gene and Δ*amrZ* mutants have an RSCV phenotype and elevated levels of c-di-GMP (Figure 1B), we hypothesized that *PA4843* encoded a diguanylate cyclase that is de-repressed in Δ*amrZ* mutants. To address this, *PA4843* was cloned into the arabinose inducible vector pHERD20T [61] and the plasmids transferred to wild type PAO1 or a strain lacking *PA4843*. c-di-GMP levels in both PAO1 or *ΔPA4843* containing the induced vector control exhibited low levels of c-di-GMP (∼3 fmol/µg total protein) (Figure 5). However, expression of *PA4843* in these strains generated nearly thirty fold more c-di-GMP (87 fmol/µg total protein for PAO1, and 92 fmol/µg total protein for Δ*PA4843*; Figure 5), supporting the hypothesis that *PA4843* is a functional diguanylate cyclase. Based on these results and others below, we named *PA4843 adcA*, for AmrZ-dependent cyclase A. Additionally, a deletion of *adcA* in a Δ*amrZ* mutant returns the c-di-GMP to wild-type levels, (Δ*amrZ* mutant 7.33 fmol/µg total protein, Δ*amrZ* Δ*adcA* double mutant 2.33 fmol/µg total protein) indicating that the elevated c-di-GMP in a Δ*amrZ* mutant is dependent on AdcA.

### The Δ*amrZ* mutant hyper biofilm phenotype is *adcA*- and c-di-GMP-dependent

**Figure 6:**
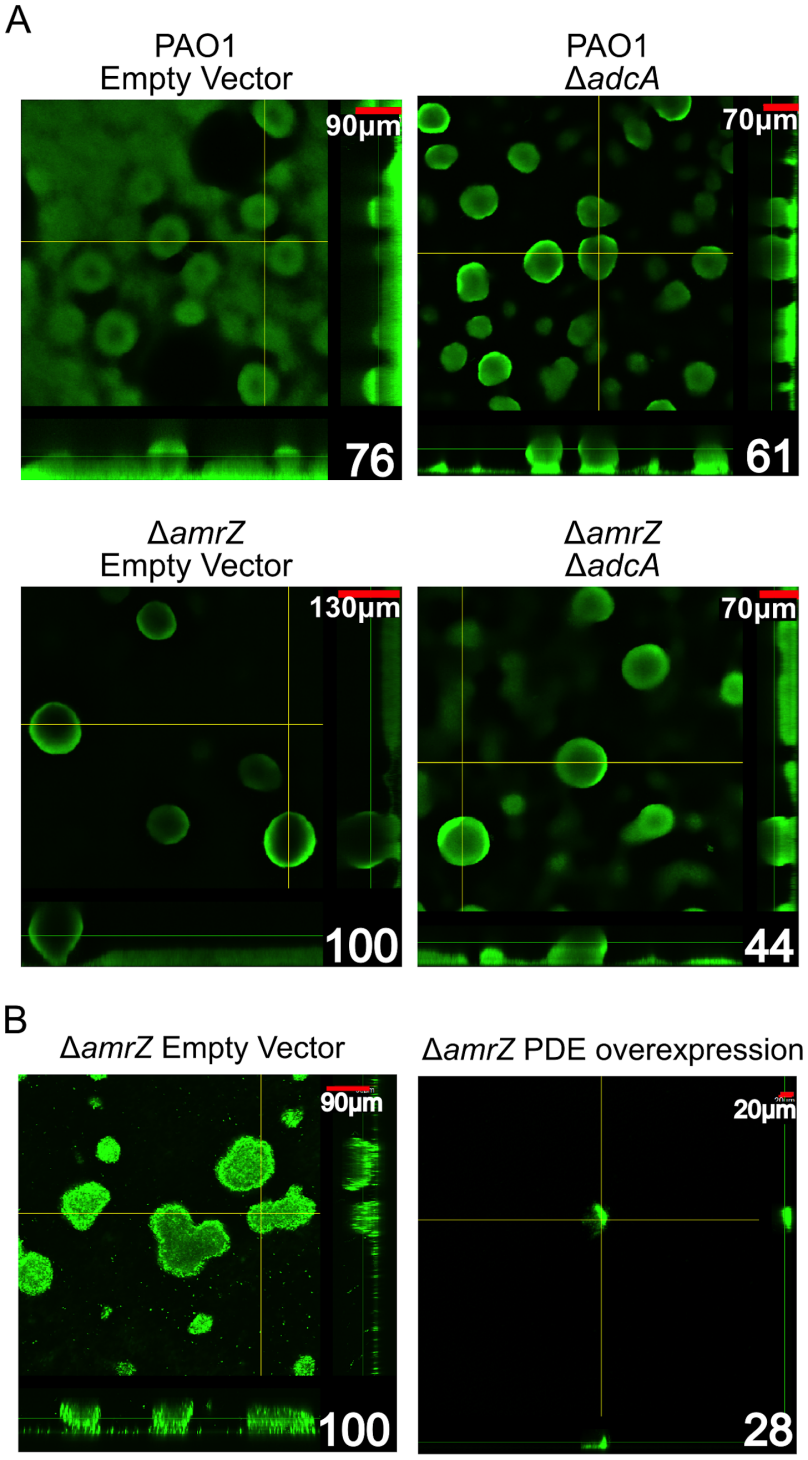
The Δ*amrZ* mutant strain hyper biofilm phenotype is *adcA* dependent. A. Biofilm biomass and microcolony height correlate with *adcA* expression. Orthogonal confocal laser scanning microscopy images of 24-hour flow cell biofilms are shown with Syto-9 stained biomass depicted as green. Scale bars are in the upper right corner of each image and percent biomass (determined via COMSTAT analysis) relative to the Δ*amrZ* empty vector are indicated in the lower right corner. Significance was determined with Student's *t*-test. (* p≤0.05). B Expression of a phosphodiesterase abrogates the Δ*amrZ* biofilm phenotype. Orthogonal confocal laser scanning microscopy images of 16-hour flow cell biofilms are shown with Syto-9 stained biomass depicted as green. Scale bars are in the upper right corner of each image and percent biomass (determined via COMSTAT analysis) relative to the *ΔamrZ* empty vector are indicated in the lower right corner. Biofilms were initiated and grown in the presence of 0.5% arabinose to induce expression of the phosphodiesterase PA2133 from the plasmid pJN2133. Significance determined with Student's *t*-test. (* p≤0.05)

As previously reported, a Δ*amrZ* mutant forms robust biofilms with more biomass and taller microcolonies than the parental strain, PAO1 [19]. This report demonstrated that direct repression of the *psl* operon by AmrZ could abrogate the hyper biofilm phenotype of the Δ*amrZ* mutant [19]. Here, we present data that AmrZ also regulates c-di-GMP concentrations in the cell, thus providing an additional level of control. We hypothesized that the Δ*amrZ* mutant hyper biofilm phenotype is due to *adcA* derepression and c-di-GMP accumulation in this strain. To test this hypothesis, we grew 24-hour flow cell biofilms in a PAO1 and Δ*amrZ* mutant background while modulating the amount of *adcA* expression in the cells (Figure 6A). We reasoned if the hyper biofilm phenotype is dependent on derepression of *adcA* and accumulation of c-di-GMP in the Δ*amrZ* mutant, biofilm cells formed by a Δ*amrZ* Δ*adcA* double mutant should have less intracellular c-di-GMP and biofilms with less biomass and microcolony height. Consistent with this hypothesis, the Δ*amrZ* Δ*adcA* double mutant produces biofilms with significantly less biomass than the Δ*amrZ* mutant (Figure 6A, Figure S3). Additionally, we observed that the Δ*amrZ* Δ*adcA* double mutant produced significantly lower c-di-GMP compared to the Δ*amrZ* mutant (2.33 vs 5.98 fmol/µg total protein, respectively) while the *adcA* overexpressing Δ*amrZ* mutant had significantly higher c-di-GMP (67.00 fmol/µg total protein). These data indicate that the hyper biofilm phenotype of the Δ*amrZ* mutant is due to loss of repression of *adcA* and elevated intracellular c-di-GMP. This mechanism, in addition to the previously reported direct repression of the *psl*-encoded biofilm polysaccharide [19], indicates that AmrZ-dependent regulation of the *psl* operon at multiple levels may amplify the effect on Psl production, with significant changes in the biofilm phenotype.

We also reasoned that if dysregulation of c-di-GMP production is responsible for the hyper biofilm phenotype of Δ*amrZ* mutants, then reducing intracellular c-di-GMP in these strains by overexpressing a phosphodiesterase (PDE) should ablate biofilm formation. For this, a plasmid encoding the arabinose inducible PDE *PA2133* (pJN2133) or the empty vector pHERD20T was transformed into the Δ*amrZ* mutant and 16 hour flow cell biofilms were grown in the presence of inducer [32]. CLSM analysis demonstrates that PDE overexpression significantly reduces biofilm biomass and microcolony height in these biofilms (Figure. 6B). These data further support the hypothesis that the hyper biofilm phenotype of Δ*amrZ* mutants is dependent on elevated c-di-GMP.

## Discussion

Understanding how bacteria respond to varying conditions in the environment and during infection is clearly of importance. Here, we present a comprehensive analysis of a bacterial transcription factor regulon obtained by combining ChIP-Seq and RNA-Seq. The power of these techniques stems from the unbiased and genome-wide production of the entire regulon, but also the activity of the transcription factor at these binding sites. These techniques have been established in eukaryotes [62], [63], however they have recently been adapted as powerful tools to investigate the activity of bacterial transcription factors [27], [28], [58], [64]–[67]. We were able to identify 398 regions bound by AmrZ in the *P. aeruginosa* genome. Additionally, we developed a transcriptional profile of both the Δ*amrZ* mutant and its complemented strain. This allowed us to combine the results of ChIP-Seq and RNA-Seq and divide loci into several categories, either activated, repressed, or unaffected by AmrZ. Each of these groups were then further categorized into directly or indirectly regulated.

Our prior studies revealed that wild type bacteria have a competitive advantage over Δ*amrZ* mutant bacteria in a mixed acute pulmonary model of infection [18]. By combining ChIP-Seq and RNA-Seq analysis, we identified many genes that are AmrZ-regulated and may be important for colonization and disease progression. One of the directly AmrZ-repressed genes, a diguanylate cyclase we named *adcA* (*PA4843*), emerged as the most highly regulated AmrZ target. Deletion of *adcA* in a Δ*amrZ* mutant eliminated the accumulation of c-di-GMP and the hyper biofilm phenotype. The modulation of c-di-GMP by AmrZ is a novel observation and enhances the molecular explanation for the earlier studies regarding the role of AmrZ in biofilm phenotypes [19]. c-di-GMP has diverse functions in *P. aeruginosa*, regulating polysaccharide production, motility, virulence factor production, and biofilm formation [60], [68]. When competed against the wild type PAO1 in an acute pulmonary infection model, both a Δ*adcA* mutant and a Δ*amrZ* Δ*adcA* double mutant retained the virulence defect observed for the Δ*amrZ* mutant (Figure S2). We propose that AmrZ-dependent gene regulation is most important in the establishment of chronic infections, as in the cystic fibrosis lung. Therefore, lack of a phenotype in an acute model of infection does not negate a role for AmrZ in chronic infections and future studies are geared towards this line of investigation. It should be noted that suitable chronic lung infection models that faithfully reproduce CF pathology are limited, though there are several very promising developments in this area [69].

Regulation of the numerous DGC and PDE enzymes in *P. aeruginosa* presents a complex network of integrated stimuli sensation and physiological response. Work in other systems has demonstrated that c-di-GMP is freely diffusible in the cytoplasm and is detected by many sensors [31], [70], [71]. This work highlights the regulation of one DGC, however, deciphering the regulation of c-di-GMP production and cellular response to diverse signals is currently an area of great interest.

In addition to the DGC activity described here, AdcA contains a predicted N-terminal two-component receiver domain. This combination of receiver domain and DGC is also observed in the well-characterized PleD of *C. crescentus* [72], [73]. Previous studies have revealed PleC-dependent activation of the PleD receiver domain by phosphorylation, leading to dimerization and c-di-GMP production [72]. The end result of this signaling cascade is the loss of flagellum and development of the stalk leading to a sessile lifestyle. Another example of a hybrid response regulator/diguanylate cyclase with biofilm effects is WspR of *P. aeruginosa* [32]. Surface growth leads to phosphorylation of WspR, inducing clustering of the protein and activation of cyclase activity [74], [75]. This model of clustered cyclases suggests that such subcellular foci can lead to regional increases of c-di-GMP, which may be an explanation for why subtle changes in whole-cell c-di-GMP pools can have drastic and varied effects on biofilm and motility phenotypes [73]–[76]. Analysis of AdcA for conserved domains indicates that the aspartate at residue 300 is a probable phosphorylation site. Activation of AdcA in *P. aeruginosa* leads to a hyper biofilm phenotype, suggesting that AdcA, PleD, and WspR have similar cellular effects. Based on the homology between these proteins, future studies will identify the partner sensor kinase and evaluate the effects of AdcA phosphorylation.

**Figure 7:**
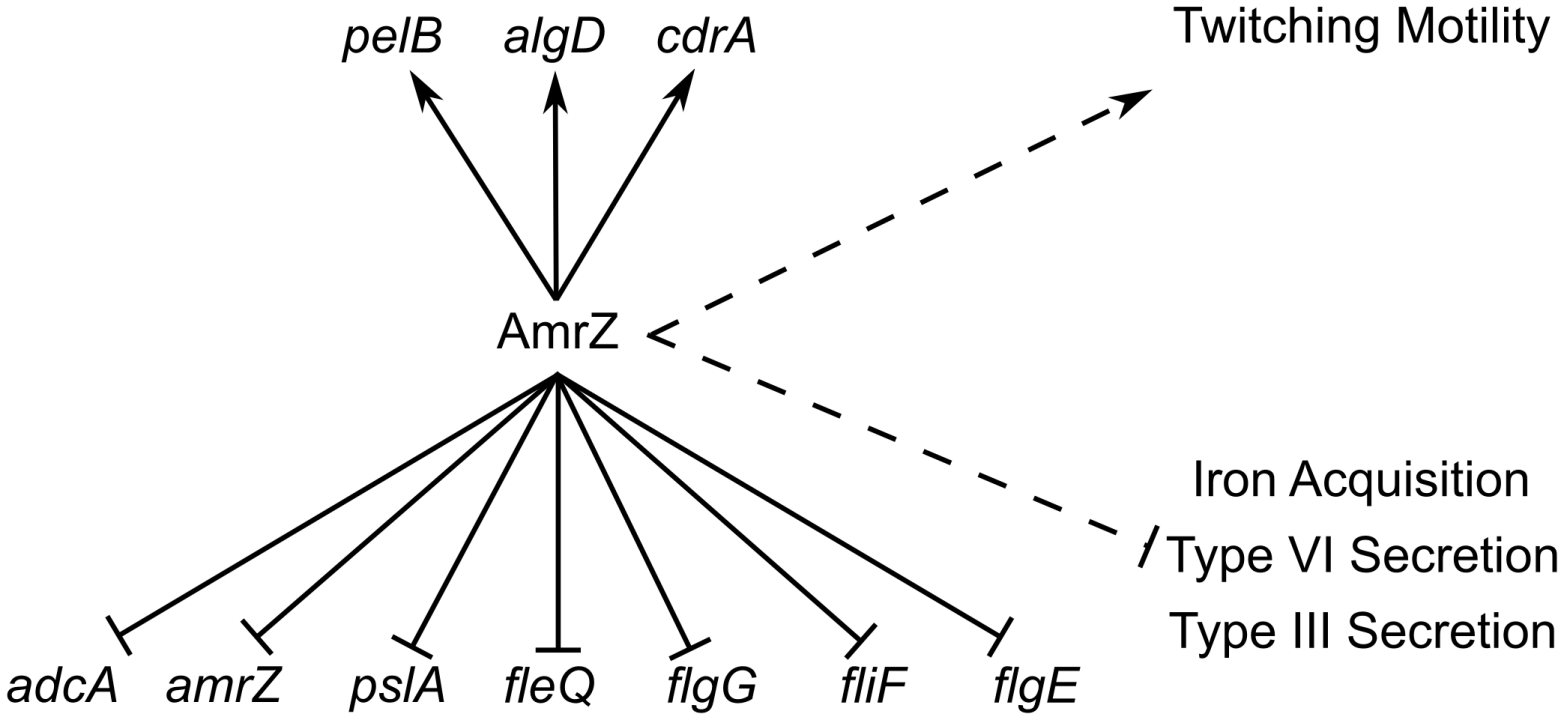
ChIP-Seq and RNA-Seq establish the AmrZ regulon. Direct (solid lines) and indirect (dashed lines) activity of AmrZ on various genes involved in virulence. Current work has expanded the understanding of the AmrZ regulon to include several new systems, including iron acquisition, type III secretion, type VI secretion, flagellum structure, Pel polysaccharide, and the biofilm structural protein CdrA.

AmrZ activates alginate transcription and twitching motility, but represses Psl, flagella, and c-di-GMP production (Figure 7). Each of these pathways have been implicated in biofilm formation and disease chronicity [77]–[85]. The complete analysis of the AmrZ regulon indicates that AmrZ may serve as a molecular switch that triggers biofilm maturation in *P. aeruginosa*. We have observed that nonmucoid, environmental strains produce a low amount of AmrZ, allowing for high production of the adherent and aggregative polysaccharide Psl [19]. Additionally, low AmrZ in these strains allows expression of *fleQ* and flagellum production, further enhancing the attachment phenotypes [21], [80]. We present here that low AmrZ also permits expression of the diguanylate cyclase *adcA*, producing elevated c-di-GMP in the cell. This signaling molecule can affect the production of all of the above pathways in addition to the direct regulation by AmrZ [29], [32], [37], [38]. Cumulatively, the result of de-repression of these genes results in a motile strain that is primed to colonize and form biofilms by expressing the adhesive polysaccharide Psl. We observe a hyper aggregative and hyper biofilm phenotype in the Δ*amrZ* mutant, supporting this hypothesis. A similar phenomenon is observed in a Δ*retS* mutant, where elevated c-di-GMP leads to hyper biofilm formation [40], [86]. The GacS/RetS sensor systems are involved in the transition from acute to chronic infections by regulating polysaccharide production, motility, and secretion systems [40], [86], [87]. These systems regulate virulence genes through RsmA, which has a vast regulon [41], [88]. Though AmrZ was not identified as regulating any of the members of the Gac/Rsm signaling cascade, the ultimate effects of the two pathways are strikingly similar. Further work will investigate how AmrZ is interacting or overlapping with these well-established regulators of acute to chronic transition. Identification of the signal activating AdcA will enhance the understanding of the interactions of these two functionally similar pathways.

Strains of *P. aeruginosa* that infect patients are Psl-producing, nonmucoid, and form biofilms more readily than mucoid strains [79], [89]. Once a cystic fibrosis patient is infected with a nonmucoid strain, there is an aggressive neutrophil influx into the lungs [90]. These neutrophils produce many antimicrobial products, including reactive oxygen species, antimicrobial peptides, and neutrophil nets [91], [92]. Additionally, CF patients with active infections are treated with high doses of antibiotics. These factors, coupled with the high salinity, low oxygen, and high viscosity of the mucus in the CF lung, provide an environment that is highly selective for bacterial variants able to persist [93]. One clear phenotype that emerges in this environment is the production of alginate (mucoidy), which provides resistance to phagocytosis and protection against antibiotics and reactive oxygen species [7], [16], [94]–[96]. Mucoid strains express AmrZ at levels much higher than those observed in nonmucoid counterparts [24], [25], [43]. We propose that AmrZ acts as a molecular switch that transitions *P. aeruginosa* from a motile, adherent, colonizing strain causing acute virulence and tissue damage to a nonmotile, mucoid, chronic strain that is more adept at persistence and immune evasion. We suggest that the enhanced virulence of the wild type is due to the expression of various virulence factors such as the type III secretion system regulator ExsC and iron sequestration proteins such PchC and FptA. AmrZ represses these genes (−3.4, −9.01, and −5.45-fold, respectively), though their promoters were not identified as bound by AmrZ in the ChIP-Seq analysis, suggesting that this repression is indirect. When AlgT/U is active, as in mucoid strains, the amount of AmrZ rises. This rise in AmrZ could reduce production of these proteins and limit the acute virulence of the strains, allowing for the establishment of a chronic infection. Additionally, we demonstrate that AmrZ activates expression of *cdrA*, encoding a biofilm matrix protein and the *pel* polysaccharide operon. Previous reports indicate that AmrZ can directly repress the *psl* operon, leading to multifactorial control of this polysaccharide [19]. Combined with the published knowledge of the effect of c-di-GMP on the *psl* operon through FleQ, these data further reinforce the potential for additive effects of AmrZ at multiple points of polysaccharide and matrix protein regulation. Cumulatively, these experiments suggest that the high production of AmrZ in mucoid strains during chronic infections could lead to a polysaccharide transition from expressing Psl to alginate and Pel. Additionally, CdrA has been reported to stabilize biofilm structure [30]. The overlap of these regulatory networks with the inclusion of c-di-GMP signaling could provide insight to the complexity of the contributions of polysaccharides to virulence during different stages of infection. Future work will delve into virulence contribution by the AmrZ-regulated genes to identify the molecular basis for the acute virulence defect in the Δ*amrZ* mutant.

## Materials and Methods

### Ethics statement

All animals were maintained in the OSU College of Medicine IACUC-approved vivarium located in the Biomedical Research Tower. The University has many veterinarians and trained animal caretakers available for consultation on the studies. The protocol for these studies has been approved by the OSU IACUC committee (Protocol # 2009A0177). There is adequate space for the animals to be housed in the vivarium. Animals are monitored frequently during the infection. Animals that meet the criteria for removal from study will be euthanized via CO_2_ inhalation. Each room contains sentinel mice that are sacrificed at regular time points for examination for infectious agents by vivarium staff. During infection, mice were lightly sedated with isoflurane and inoculated intranasally with bacteria suspended in sterile PBS. Thirty µL of the PBS solution is pipetted onto the nares of the mouse as soon as the anesthetic administration is discontinued. The animal rapidly recovers under supervision from the researcher. The mice are not in discomfort or distress during this procedure. There are no restraining devices utilized during this study. Mice were sacrificed via CO2 inhalation. This method of euthanasia causes minimal discomfort to the animals. Cardiac puncture was used as a second method of euthanasia. These methods are consistent with the recommendations of the American Veterinary Medical Association Guidelines on Euthanasia.

### Bacterial strains and growth conditions

The bacterial strains used along with genotypes are provided in Table S3. *P. aeruginosa* strains were inoculated in LBNS (10 g l^−1^ tryptone, 5 g l^−1^ yeast extract, pH 7.5) at 37°C for overnight cultures under shaking conditions unless otherwise noted. Strains were grown at 37°C on LANS (LBNS with 1.5% agar) or *Pseudomonas* Isolation Agar (Difco, Detroit, MI) agar plates. *E. coli* was routinely cultured at 37°C in lysogeny broth (LB, 10 g L^−1^ tryptone, 5 g L^−1^ yeast extract, 5 g L^−1^ NaCl). Semi-solid media was prepared by adding 1.5% Bacto agar to LB. Colony morphology was imaged on modified Vogel-Bonner minimal medium (VBMM) plates (0.2 g L^−1^ MgSO_4_ 7H_2_O, 2.0 g L^−1^ citric acid, 3.5 g L^−1^ NaNH_4_HPO_4_ 4H_2_O, and 10 g L^−1^ K_2_HPO_4_, 1 g L^−1^ casamino acids, and 5 mM CaCl_2_. Congo Red (40 µg/mL) and Brilliant Blue R (15 µg/mL) were added to VBMM to aid in visualization of morphology. Antibiotics were added to maintain or select for plasmids in *P. aeruginosa* as follows: gentamicin (Gm) at 100 µg/mL, Rifampicin (Rif) at 100 µg/mL and carbenicillin (Cb) at 300 µg/mL. Antibiotics were added to maintain or select for plasmids in *E. coli* as follows: gentamicin (Gm) at 10 µg/mL and spectinomycin (Sp) at 50 µg/mL.

### Plasmid construction

Plasmids and primers used in genetic manipulations are listed in Tables S4 and S5, respectively.

Primers AmrZF2 and AmrZR2 amplified the 324 bp DNA sequence of *amrZ*. NEB Q5 High Fidelity DNA Polymerase was used in PCR following manufacturer's instructions. The PCR product of *amrZ* was inserted into pET29a (Novogen) through *Nde*I and *NotI* restriction sites. The 432 bp DNA sequence of the *amrZ* gene, ribosome binding site, and C-terminal 6x His tag were amplified from the resulting plasmid using primers AmrZF3 and AmrZR3. The PCR product was inserted into pHERD20T [61] through *Xba*I and *Hin*dIII restriction sites. The resulting construct (pCJ3) was verified by DNA sequencing.

A deletion allele for *PA4843* was assembled by removing an in-frame, 1593 bp fragment of coding sequence from the *PA4843* open reading frame (ORF), leaving a scar ORF encoding a 10-amino acid peptide. In a first step, two PCR products were amplified using primers that targeted the adjacent upstream and downstream regions of the chromosome flanking *PA4843*. Subsequently, these PCR products were joined by splicing by overlapping extension (SOE) PCR [97] to create the Δ*PA4843* allele. The upstream forward and downstream reverse primers used to generate this deletion allele were tailed with *attB*1 or *attB*2 sequences as described in the Gateway Cloning Technology Manual (Invitrogen). Using Gateway technology, the Δ*PA4843* allele was first recombined with pDONR223 using BP Clonase II (Invitrogen) to create pJJH125, which was sequenced using M13F and M13R primers. Finally, the Δ*PA4843* allele from pJJH125 was recombined with pEX18GmGW using LR Clonase II (Invitrogen) to create the allelic exchange vector pJJH129.

The *adcA* overexpression plasmid pBX22 was constructed by inserting *adcA* coding sequence into the arabinose-inducible vector pHERD20T [61]. The 1659 bp DNA sequence of the *adcA* gene was amplified by primers PA4843_F and PA4843_R. NEB Q5 High Fidelity DNA Polymerase was used in PCR following manufacturer's instructions. The PCR product of *adcA* was inserted into pHERD20T through XbaI and HindIII restriction sites. The *adcA* coding sequence in pBX22 was verified by Sanger-based DNA sequencing.

### Quantification of c-di-GMP by LC/MS

c-di-GMP was extracted and quantified as described previously with minor modifications [39]. Cells were cultured overnight on LANS plates. An isolated colony was transferred to a fresh LANS plate and incubated at 37°C for 24 hrs before harvesting. Colonies were scraped from agar plates and resuspended in 990 µL of LC/MS grade water (Optima). 2-chloro-adenosine-5′-O-monophosphate (2Cl-AMP, 10 µL of 10 µM, Biolog), was added as an internal standard. Nucleotides were extracted from cells by the addition of 94 µl of 70% perchloric acid and incubated for 30 min on ice. Cell debris were removed by centrifugation and reserved for subsequent protein quantification. The supernatant containing c-di-GMP was neutralized by the addition of 219 µL of 2.5 M KHCO_3_. The resulting precipitate was removed by centrifugation. The supernatant was stored at -80°C until LC/MS analysis. Pure c-di-GMP standards (Biolog) were extracted in parallel and treated identically to samples.

Compounds were separated on an Acuity UPLC equipped with a C18 Guard Cartridge (Phenomenex) and Synergi 4 µ Hydro RP 80A column (50×2 mm, Phenomenex). The injection volume was 20–30 µL. A gradient was established starting with 98% aqueous (10 mM formic acid in water) and 2% organic (acetonitrile). The aqueous concentration was adjusted to 70% at 2 min, 20% at 2.5 min, 100% at 3 min, and finally held at 98% from 5–7.5 min. Compounds were detected using multiple reaction monitoring on a Premier XL triple-quadrupole electrospray mass spectrometer (Waters) in positive-ionization mode. The m/z 691>152 transition was used for the identification of c-di-GMP and 382>170 for 2Cl-AMP. The cone voltages and collision energies were 40 V/30 eV and 35 V/20 eV, respectively. The capillary voltage used was 3.5 kV. The desolvation temperature was 350°C and source temperature was 120°C. Nitrogen was used as a drying gas with a flow rate of 800 L/hr. The concentrations of c-di-GMP were calculated by comparison of the peak area ratio of c-di-GMP to 2Cl-AMP to a standard curve. Moles of c-di-GMP were normalized to total protein determined from Pierce protein assay. Data represent averages of three independent cultures.

For protein quantification, cell pellets were resuspended in 220 µL of 10 mM Tris-Cl buffer (pH 8.5). The remaining acid in the pellets was neutralized by the addition of 30 µl of 1 M NaOH. Cells were lysed by the addition of 250 µl of 2X concentrated Laemilli Buffer and boiled for 30-90 min at 100°C or until the pellet had dissolved. Protein concentration was determined using Pierce 660 nm Protein Reagent with Ionic Detergent Compatibility Reagent (IDCR) as recommended by the manufacturer.

### Chromatin immunoprecipitation

Chromatin immunoprecipitation was modified from existing protocols [44], [45]. Cultures were induced with 0.5% arabinose at an OD_600_ of 0.1 and allowed to grow for two hours at 37°C in a roller. Protein-DNA complexes were cross-linked by addition of formaldehyde- to a final concentration of 1.0% and incubated at room temperature for ten minutes. Cross-linking was quenched by addition of glycine (final concentration 250 mM). The final OD_600_ was recorded and cells were collected from 1 OD_600_ of culture via centrifugation and washed once in LBNS. The supernatant was removed and pellets were stored for further processing at -80°C.

Cell pellets were resuspended in 1.0 mL of lysis buffer (20 mM HEPES, pH 7.9; 50 mM KCl; 0.5 mM DTT; 500 mM NaCl; 10 mM imidazole; 1% BSA; 1 µg/mL leupeptin/pepstatin; and 400 µM PMSF) per 1 OD_600_ of culture. Samples were sonicated on Covaris with the following conditions: Duty Cycle 20%, Intensity 8, Cycles per burst 200, with frequency sweeping 20 min total shearing time (60 sec cycles, 20 cycles). Lysate was cleared via centrifugation (20,000× g, 30 minutes, 4°C) and the supernatant was transferred to a fresh tube as the input sample. Magne-HIS beads (Promega V8560) were blocked at room temperature in lysis buffer for 30 minutes, and then 500 µl of the input sample was added to the beads. After 30 minutes of binding at room temperature with agitation, the supernatant was removed from the beads via magnetic separation. Beads were washed five times in wash buffer (100 mM HEPES, pH 7.5, 10 mM imidazole, 500 mM NaCl, and 1% BSA). Elution buffer (100 mM HEPES, pH 7.5; and 500 mM imidazole) was added to the beads and incubated at room temperature for 30 minutes. Supernatant was collected after magnetic separation and combined with SDS (1.25% final concentration), then heated to 70°C for 30 minutes to reverse cross-links. DNA was purified via phenol:chloroform extraction and ethanol precipitation [98].

### ChIP-Seq library construction and sequencing

The chip DNA was quantified with Qubit 2 flurometer (Life Technologies) using Qubit dsDNA BR Assay. 10 ng of DNA was used to construct each Chip sequencing library, following NEXTflex ChIP-Seq kit (Bioo Scientific) instruction. NEXTflex ChIP-Seq Barcodes (Bioo Scientific) were used to index the library. The final DNA libraries were validated with Agilent 2100 Bioanalyzer using Agilent High Sensitivity DNA Kit. And the library concentrations were determined by Q-PCR using KAPA SYBR Fast qPCR kit. The libraries were then run on Single End flowcell on HiSeq2000.

### ChIP-Seq data analysis

HiSeq2000 sequencing was performed, resulting in approximately 255 million total single-end 52 bp reads from the six control and eight treatment samples. Reads were aligned using bwa (0.5.10) to the *Pseudomonas aeruginosa* PAO1 reference genome [99]. Approximately 220 million reads aligned uniquely to the reference (86.3%). A TDF file was created for each sample for visualization in IGV, which was scaled to reads per 10 million data using bedtools (2.17.0) and igvtools (2.3.3). ChIP-Seq analysis was performed using HOMER (4.2). First, aligned data was transformed into a platform-independent data structure for further HOMER analyses using the *makeTagDirectory* function. Secondly, HOMER's *findPeaks-style factor* was utilized to identify peaks, or regions of the genome where more reads are present than random. Lastly, HOMER's *findMotifsGenome.pl* was used to analyze genomic positions for *de novo* enriched motif regions of length 50 or 200 and identified peaks were annotated with the motifs using the *annotatePeaks.pl* function.

### RNA isolation

Cultures were induced with 0.5% arabinose at an OD_600_ of 0.1 and allowed to grow for two hours at 37°C in a roller. The final OD_600_ was recorded and 0.1 OD_600_ was centrifuged at 10,000-x g for 3 minutes. The supernatant was removed and pellets were resuspended in 1 mL of TRIzol (Invitrogen). Following a 5-minute incubation at room temperature, 0.2 mL of chloroform was added and the samples were shaken for 15 minutes. Phases were separated by centrifugation (12,000× g, 5 minutes, 4°C) and the aqueous phase was combined with 0.6 mL of 70% ethanol and transferred to an RNeasy mini column (Qiagen). After centrifugation, 0.7 mL of buffer RW1 (Qiagen) was added to the column and centrifuged. Samples were washed twice with 0.5 mL of Buffer RPE (Qiagen) and eluted in 50 µL of water.

### RNA-Seq library construction and sequencing

Following assessment of the quality of total RNA using Agilent 2100 bioanalyzer and RNA Nano Chip kit (Agilent Technologies, CA), rRNA was removed from 2.5 µg of RNA with Ribo-Zero rRNA removal kit for Gram-negative bacteria (Epicentre Biotechnologies, WI). To generate directional signal in RNA seq data, libraries were constructed from first strand cDNA using ScriptSeq v2 RNA-Seq library preparation kit (Epicentre Biotechnologies, WI). Briefly, 50 ng of rRNA-depleted RNA was fragmented and reverse transcribed using random primers containing a 5′ tagging sequence, followed by 3′end tagging with a terminal-tagging oligo to yield di-tagged, single-stranded cDNA. Following purification by a magnetic-bead based approach, the di-tagged cDNA was amplified by limit-cycle PCR using primer pairs that anneal to tagging sequences and add adaptor sequences required for sequencing cluster generation. Amplified RNA-seq libraries were purified using AMPure XP System (Beckman Coulter). Quality of libraries were determined via Agilent 2100 Bioanalyzer using DNA High Sensitivity Chip kit, and quantified using Kappa SYBRFast qPCR kit (KAPA Biosystems, Inc, MA). 50 bp sequence reads were generated using the Illumina HiSeq 2000 platform.

### RNA-Seq data analysis

HiSeq 2000 sequencing was performed, resulting in approximately 165 million total single-end 52-bp reads from the six total control and treatment samples. Reads were aligned using bwa (0.5.10) to the *P. aeruginosa* PAO1 reference genome [99]. Approximately 143 million reads aligned uniquely to non-ribosomal regions of the reference (86.9%). A TDF file was created for each sample for visualization in IGV, which was scaled to reads per million data using bedtools (2.17.0) and igvtools (2.3.3). A coverage file, describing the coverage for each feature in the PAO1 genome, was created using bedtools. These coverage's were normalized and the means of the control and treatment groups were tested for significant differences using the binomial test in the R package DESeq (1.10.1), producing fold changes and adjusted p-values for each feature. Resulting p-values were adjusted for multiple testing with the Benjamin-Hochberg procedure, which controls false discovery rate (FDR).

### EMSA

6FAM labeled DNA used for EMSA was amplified using Quick-load Taq 2X Mastermix (New England Biolabs), FAM-labeled forward primer and non-labeled reverse primer, and PAO1 genomic DNA as the template. The EMSA procedure is similar to that previously reported [18]. Each EMSA reaction contains 4 mM Tris-HCl (pH8.0), 40 mM NaCl, 4 mM MgCl2, 4% glycerol, 150 ng/ul Poly d[(I-C)] (non-specific DNA control), 100 µg/mL BSA (non-specific protein control), 5 nM FAM labeled DNA, and a defined concentrations of AmrZ or AmrZR22A. Protein-DNA binding was equilibrated at room temperature (25°C) for 20 min after adding all reagents to each reaction. 10 µL of each reaction was loaded onto a 4% non-denaturing acrylamide gel. Electrophoresis was conducted for 22 min at 200 V in 0.5% TBE. 6FAM fluorescence was detected with a Typhoon scanner (GE Lifescience). A similar length DNA sequence within the *algD* coding sequence but lacking an AmrZ binding site was selected as the specificity control.

### Protein structure and function prediction

Protein sequence was submitted to the Phyre2 server for analysis of homology [100]. Predicted structure was imaged in Jmol (http://www.jmol.org).

### Flow cell biofilm study

Inoculation of flow cells was done by normalizing overnight cultures to an optical density of 0.5 and injecting into an Ibidi μ-Slide VI^0.4^ (Ibidi 80601). To seed the flow cell surface, the media flow was suspended and the bacteria allowed to adhere at room temperature for 3 hours. Flow of 5% v/v LBNS with 0.5% arabinose was initiated at a rate of 0.15 mL*min^−1^ and continued for 24 h. Following the biofilm growth period, the flow was terminated and the biofilms were fixed with 4% paraformaldehyde. Confocal images were taken at the Ohio State University Campus Microscopy and Imaging Facility on an Olympus Fluoview 1000 Laser Scanning Confocal microscope. Images were obtained with a 20X oil immersion objective. Images were processed using the Olympus FV1000 Viewer software. Quantitative analyses were performed using the COMSTAT software package [101] Total biomass was determined from Z-stack images using the BIOMASS command with the threshold set to 15. Three independent biofilms were imaged and analyzed. Statistical significance was determined using a Student's t-test.

## Supporting Information

Figure S1 **A Phyre 2 structural model of AdcA.** Structural model of AdcA is based on similarities to PleD of *C. crescentus*. Model is colored based on amino acid position, with the N-terminus red and the C-terminus violet. Predicted cyclase active site (GGEEF), allosteric inhibitory site (I-site), and two component receiver domains (Rec domains) are indicated.
(TIFF)

Figure S2 **AdcA is not responsible for the acute virulence defect of a** ***ΔamrZ*** **mutant.** Strains were coinoculated intranasally at a 1∶1 ratio (10^8^ total bacteria). Lungs were harvested, homogenized, and plated for CU at 24 hours post infection. Competitive index is displayed, comparing the input ratio of bacteria to the output ratio. Mean of the competitive index of three independent experiments (n = 5) are displayed, comparing input ratio of bacteria to output ratio of bacteria. Groups were compared using the Student's *t*-test. ns = No significant difference.
(TIFF)

Figure S3 **Quantification of biofilm changes using COMSTAT.** Average total biomass of three biofilm images was quantified for strains PAO1, *ΔamrZ/*pBAD*amrZ*, and *ΔamrZ* strains using COMSTAT. Student's t-test was performed to determine statistical differences among these strains (* p<0.05). Unmarked comparisons are not statistically significant.
(TIFF)

Table S1 **ChIP-Seq data.** List of all regions of the genome significantly enriched for AmrZ binding as determined by ChIP-Seq. ChIP-Seq fold enrichment indicates the change in detection of a region comparing the sequences complexed to the induced AmrZ to the input DNA from that sample. Predicted binding motif is included. The closest predicted promoter to the predicted binding site is listed. Expression fold change as determined by RNA-Seq is included for each region, if significant.
(XLSX)

Table S2 **RNA-Seq data.** List of all genes whose expression is significantly different comparing the complemented AmrZ strain to the *ΔamrZ* mutant. Negative fold change indicates that the expression was lower in the complemented strain than the mutant (repressed by AmrZ), while positive fold change indicates that the expression was higher in the complemented strain than the mutant (activated by AmrZ).
(XLSX)

Table S3 **Bacterial strains.** List of bacterial strains used in this study.
(DOCX)

Table S4 **Plasmids.** List of plasmids used in this study.
(DOCX)

Table S5 **Primers.** List of primers used in this study. *Regions of identity to the target amplicons are underlined, regions of reverse complementarity are *italicized*, and Gateway *attB*1 and *attB*2 sequences are in **bold**.
(DOCX)

Supporting Methods S1 **Methods utilized to produce Supporting Information.**
(DOCX)
