# Supplementary figures and images for: ChIP-Seq and RNA-Seq Reveal an AmrZ-Mediated Mechanism for Cyclic di-GMP Synthesis and Biofilm Development by Pseudomonas aeruginosa
Source: PLoS Pathog. 2014 Mar 6;10(3):e1003984. doi: 10.1371/journal.ppat.1003984 (PMC3946381; doi:10.1371/journal.ppat.1003984)

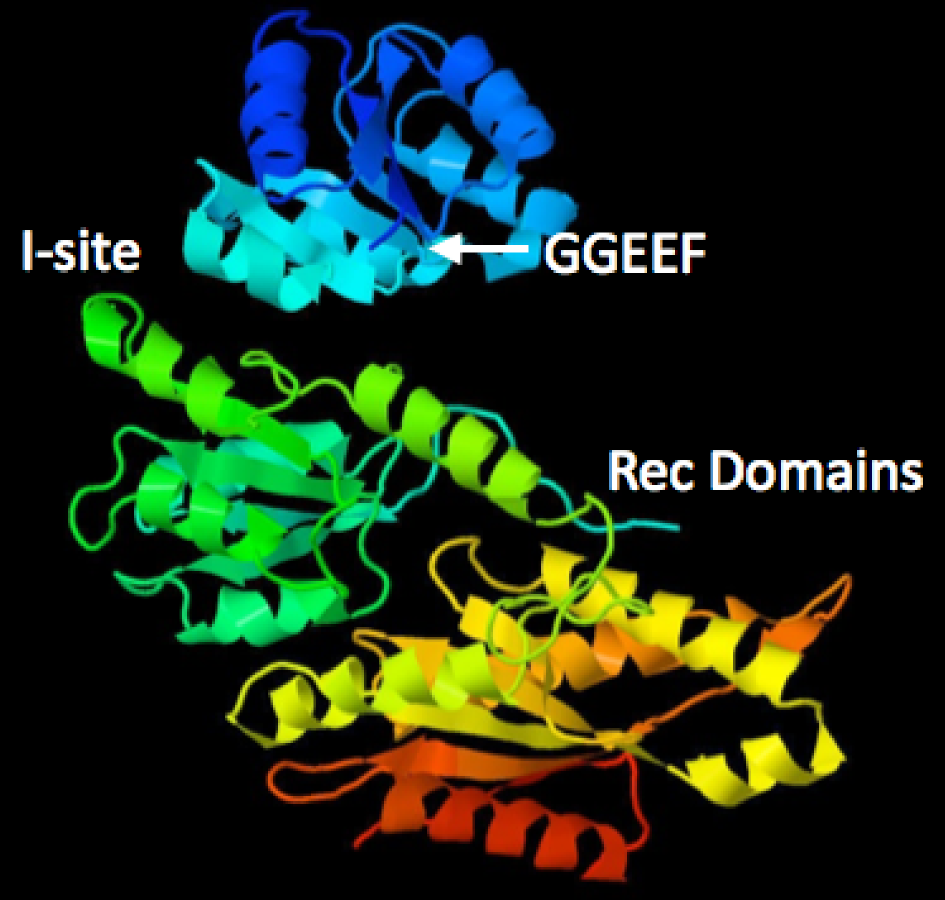

Supplement: Figure S1 — A Phyre 2 structural model of AdcA. Structural model of AdcA is based on similarities to PleD of C. crescentus. Model is colored based on amino acid position, with the N-terminus red and the C-terminus violet. Predicted cyclase active site (GGEEF), allosteric inhibitory site (I-site), and two component receiver domains (Rec domains) are indicated. (TIFF) [file ppat.1003984.s001.tif]

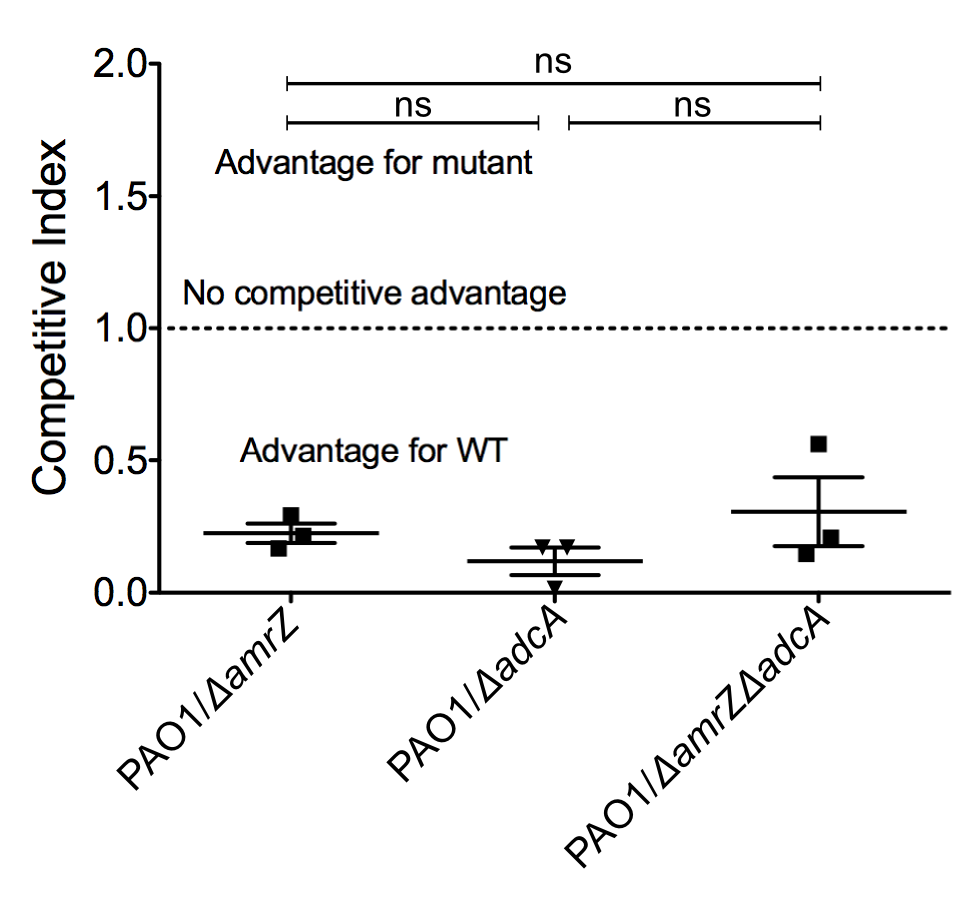

Supplement: Figure S2 — AdcA is not responsible for the acute virulence defect of a ΔamrZ mutant. Strains were coinoculated intranasally at a 1∶1 ratio (108 total bacteria). Lungs were harvested, homogenized, and plated for CU at 24 hours post infection. Competitive index is displayed, comparing the input ratio of bacteria to the output ratio. Mean of the competitive index of three independent experiments (n = 5) are displayed, comparing input ratio of bacteria to output ratio of bacteria. Groups were compared using the Student's t-test. ns = No significant difference. (TIFF) [file ppat.1003984.s002.tif]

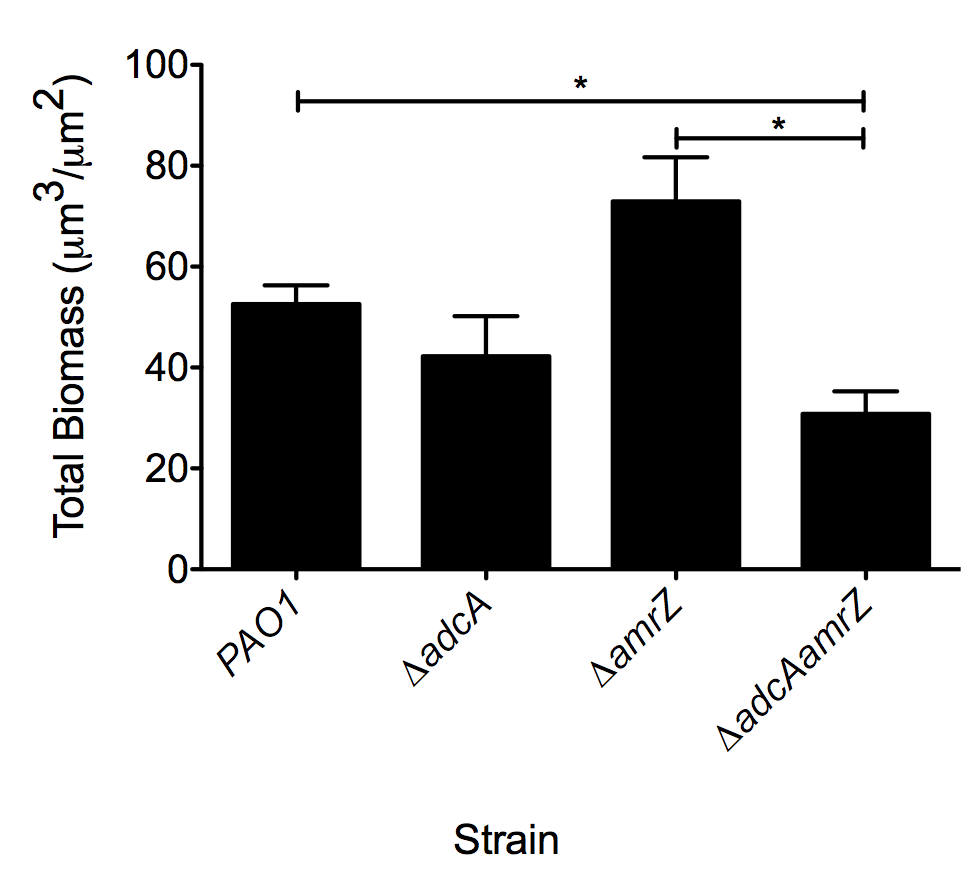

Supplement: Figure S3 — Quantification of biofilm changes using COMSTAT. Average total biomass of three biofilm images was quantified for strains PAO1, ΔamrZ/pBADamrZ, and ΔamrZ strains using COMSTAT. Student's t-test was performed to determine statistical differences among these strains (* p<0.05). Unmarked comparisons are not statistically significant. (TIFF) [file ppat.1003984.s003.tif]
