# Supplementary material for: ChIP-Seq and RNA-Seq Reveal an AmrZ-Mediated Mechanism for Cyclic di-GMP Synthesis and Biofilm Development by Pseudomonas aeruginosa
Source: PLoS Pathog. 2014 Mar 6;10(3):e1003984. doi: 10.1371/journal.ppat.1003984 (PMC3946381; doi:10.1371/journal.ppat.1003984)
Supplement: Table S3 — Bacterial strains. List of bacterial strains used in this study. (DOCX) [file ppat.1003984.s006.docx]

**Table S3. Bacterial strains.** List of bacterial strains used in this study.

| **Strain** | **Genotype, description or relevant characteristics** | **Source** |
| --- | --- | --- |
| *Escherichia coli* | | |
| ccdB Survival™ 2 T1R | F^-^ *mcrA Δ(mrr-hsdRMS-mcrBC) Φ80lacZΔM15 ΔlacX74 recA1 araΔ139 Δ(ara-leu)7697 galU galK rpsL endA1 nupG fhuA::IS2*, Sm^r^ | Invitrogen |
| NEB5α | *fhuA2 Δ(argF-lacZ)U169 phoA glnV44 Φ80Δ (lacZ)*M15 *gyrA96 recA1 relA1 endA1 thi-1 hsdR17* | New England BioLabs |
| *Pseudomonas aeruginosa* | | |
| PAO1 | Nonmucoid parental strain | [26] |
| WFPA205 | PAO1 ∆*amrZ::tet* | [26] |
| WFPA510 | PAO1 ∆*amrZ::tet, amrZ chromosomal complementation* | [26] |
| WFPA513 | PAO1 ∆*amrZ::tet, DNA binding deficient R22A amrZ chromosomal complementation* | [26] |
| OSUPA9 | PAO1 ∆*adcA* | This Study |
| OSUPA10 | PAO1 ∆*amrZ::tet* ∆*adcA* | This study |

Sm, streptomycin; Tc, tetracycline
