# Supplementary material for: ChIP-Seq and RNA-Seq Reveal an AmrZ-Mediated Mechanism for Cyclic di-GMP Synthesis and Biofilm Development by Pseudomonas aeruginosa
Source: PLoS Pathog. 2014 Mar 6;10(3):e1003984. doi: 10.1371/journal.ppat.1003984 (PMC3946381; doi:10.1371/journal.ppat.1003984)
Supplement: Table S4 — Plasmids. List of plasmids used in this study. (DOCX) [file ppat.1003984.s007.docx]

**Table S4.** **Plasmids.** List of plasmids used in this study.

| **Plasmid** | **Description or relevant characteristics** | **Source** |
| --- | --- | --- |
| pET29a | Km^r^ cloning/expression vector containing C-terminal 6x His tag | Novogen |
| pHERD20T | Cb^r^ pUCP20T P_lac_ replaced with 1.3-kb AflII-EcoRI fragment of *araC*-P_BAD_ cassette | {Qiu:2008ho} |
| pCJ3 | Cb^r^ arabinose inducible vector (pHERD20T) expressing *amrZ* (*PA3385*) with a C-terminal 6x His tag | This Study |
| pBX22 | Cb^r^ arabinose inducible vector (pHERD20T) expressing *adcA* (*PA4843*) | This Study |
| pJN105 | Gm^r^ *araC-*P_BAD_ cassette cloned in pBBR1MCS-5 | {Newman:1999tj} |
| pJN2133 | Gm^r^; *PA2133* cloned into pJN105 | {Hickman:2005ek} |
| pDONR223 | Sp^r^ and Cm^r^, entry vector for single-fragment Gateway cloning, contains the donor *attP*1/*attP*2 recombination sites flanking *ccdB* and *cat* | {Rual:2004bc} |
| pEX18GmGW | Gm^r^ and Cm^r^, allelic exchange vector, colE1 origin of replication, *oriT*_RP4_, *sacB*, containing the Gateway (GW) destination *attR*1/*attR*2 recombination sites flanking *ccdB* and *cat* | {Wolfgang:2003tl} |
| pJJH125 | Sp^r^, pDONR223 with an *attL*1/*attL*2 flanked 809 bp deletion construct for *P. aeruginosa* PAO1 *PA4843* | This study |
| pJJH129 | Gm^r^, pEX18GmGW with an *attB*1/*attB*2 flanked 809 bp deletion construct for *P. aeruginosa* PAO1 *PA4843* | This study |

Km, kanamycin; Cb, carbenicillin; Cm, chloramphenicol; Gm, gentamicin; Sp, spectinomycin.
