# Supplementary material for: ChIP-Seq and RNA-Seq Reveal an AmrZ-Mediated Mechanism for Cyclic di-GMP Synthesis and Biofilm Development by Pseudomonas aeruginosa
Source: PLoS Pathog. 2014 Mar 6;10(3):e1003984. doi: 10.1371/journal.ppat.1003984 (PMC3946381; doi:10.1371/journal.ppat.1003984)
Supplement: Table S5 — Primers. List of primers used in this study. *Regions of identity to the target amplicons are underlined, regions of reverse complementarity are italicized, and Gateway attB1 and attB2 sequences are in bold. (DOCX) [file ppat.1003984.s008.docx]

**Table S5. Primers.** List of primers used in this study. *Regions of homology to the target amplicons are underlined, regions of reverse complementarity are *italicized*, and Gateway *attB*1 and *attB*2 sequences are in **bold**.

| **Oligonucleotide** | **DNA sequence*** |
| --- | --- |
| AmrZ F2 | TTT TTT CAT ATG CGC CCA CTG AAA C |
| AmrZ F3 | TTT TTT TCT AGA AAT AAT TTT GTT |
| AmrZ R2 | TTT TTT GCG GCC GCG GCC TGG GCC AGC T |
| AmrZ R3 | TTT TTT AAG CTT AGC AGC CGG ATC T |
| PA4843_F | TGC TCT AGA CCG CGA AGA CCA CGA AAG AAG |
| PA4843_R | CCC AAG CTT CCA TTA TCG AGC GTC GGG AC |
| adcA FAM F EMSA | CGT AGT CCG TCG CAC AAA G |
| adcA R EMSA | GCG CTT CTT TCG TGG TCT TC |
| algD111 EMSA | CAA ACG GCC GGA ACT TCC CT |
| algD112 EMSA | TAG TTC GGT CCA TAG AAT TCA AG |
| JJH367_M13-Universal-F | GTA AAA CGA CGG CCA G |
| JJH368_M13-Universal-R | CAG GAA ACA GCT ATG AC |
| JJH449_PA4843upF01-GWB1 | GGG G**AC AAG TTT GTA CAA AAA AGC AGG CTC** AGT CCC ACG ACG CTT CTT C |
| JJH450_PA4843upR01 | *CAA AAG GCC ATT ATC GAG CGT CGG G*GT CAT CGT GCT CGG TCA T |
| JJH451_PA4843downF01 | CCC GAC GCT CGA TAA TGG |
| JJH452_PA4843downR01-GWB2 | GGG G**AC CAC TTT GTA CAA GAA AGC TGG GTG** GCT GAA TTC GCG CAA CTC |
