# Supplementary material for: ChIP-Seq and RNA-Seq Reveal an AmrZ-Mediated Mechanism for Cyclic di-GMP Synthesis and Biofilm Development by Pseudomonas aeruginosa
Source: PLoS Pathog. 2014 Mar 6;10(3):e1003984. doi: 10.1371/journal.ppat.1003984 (PMC3946381; doi:10.1371/journal.ppat.1003984)
Supplement: Supporting Methods S1 — Methods utilized to produce Supporting Information. (DOCX) [file ppat.1003984.s009.docx]

**Supporting Information**

**Materials and Methods**

**Acute Murine Pulmonary Infections.**  Rifampicin resistant PAO1 was isolated by plating an overnight culture of PAO1 on LANS supplemented with Rif. No growth defect was observed for the Rif resistant strain. Six week old female C57BL/6J mice were (Jackson Laboratory) acclimated for 5-7 days prior to infection. The intranasal infection was performed as previously described [18]. Briefly, mice were lightly sedated with isofluorane (Butler) and intranasally inoculated with 30μL of PBS containing 10^8^ bacteria (PAO1 and mutant in a 1:1 ratio). Animals were sacrificed and the lungs were aseptically harvested and homogenized in sterile PBS. Homogenates were serially diluted in PBS and plated on LANS for total bacterial load or LANS with Rifampicin (100μg/mL) to generate a count of wild type bacteria. Mutant bacterial counts could be generated by subtracting the count of wild type from the total bacterial load. Competitive index is the ratio of output bacteria divided by the ratio of input bacteria. Statistics were performed using an unpaired two-tailed Student’s *t-*test. All animal procedures were conducted in accordance with Ohio State University IACUC Protocol 2009A0177-R1.
